# A tamoxifen inducible knock-in allele for investigation of E2A function

**DOI:** 10.1186/1471-213X-9-51

**Published:** 2009-10-12

**Authors:** Mary E Jones, Motonari Kondo, Yuan Zhuang

**Affiliations:** 1 Department of Immunology, Duke University Medical Center, Durham, NC 27710, USA

## Abstract

**Background:**

E-proteins are transcription factors important for the development of a variety of cell types, including neural, muscle and lymphocytes of the immune system. E2A, the best characterized E-protein family member in mammals, has been shown to have stage specific roles in cell differentiation, lineage commitment, proliferation, and survival. However, due to the complexity of E2A function, it is often difficult to separate these roles using conventional genetic approaches. Here, we have developed a new genetic model for reversible control of E2A protein activity at physiological levels. This system was created by inserting a tamoxifen-responsive region of the estrogen receptor (ER) at the carboxyl end of the *tcfe2a *gene to generate E2AER fusion proteins. We have characterized and analyzed the efficiency and kinetics of this inducible E2A^ER ^system in the context of B cell development.

**Results:**

B cell development has been shown previously to be blocked at an early stage in E2A deficient animals. Our E2A^ER/ER ^mice demonstrated this predicted block in B cell development, and E2AER DNA binding activity was not detected in the absence of ligand. *In vitro *studies verified rapid induction of E2AER DNA binding activity upon tamoxifen treatment. While tamoxifen treatment of E2A^ER/ER ^mice showed inefficient rescue of B cell development in live animals, direct exposure of bone marrow cells to tamoxifen in an *ex vivo *culture was sufficient to rescue and support early B cell development from the pre-proB cell stage.

**Conclusion:**

The E2A^ER ^system provides inducible and reversible regulation of E2A function at the protein level. Many previous studies have utilized over-expression systems to induce E2A function, which are complicated by the toxicity often resulting from high levels of E2A. The E2A^ER ^model instead restores E2A activity at an endogenous level and in addition, allows for tight regulation of the timing of induction. These features make our E2A^ER ^*ex vivo *culture system attractive to study both immediate and gradual downstream E2A-mediated events.

## Background

E2A, encoded by the *tcfe2a *gene and a member of the E-protein family, is a basic helix-loop-helix (HLH) transcription factor critical for regulating gene expression in several developmental systems [1]. Originally identified as an immunoglobulin (Ig) enhancer binding factor, E2A has since largely been studied for its roles during the development of B and T lymphocytes [2,3]. E2A participates in various aspects of lymphocyte development including lineage commitment, initiation of lineage specific gene expression, rearrangement of B and T cell receptor genes, and differentiation through multiple developmental stages. In addition to roles in lineage commitment and cell differentiation, E2A has also been shown to regulate cell cycle, proliferation, and survival.

E-proteins, including E2A, HEB, and E2-2 in mammals, function as dimers to bind DNA and regulate gene expression. E2A homodimers are the primary E-protein dimers functioning in B cells, whereas E2A-HEB heterodimers are the primary dimers functioning in T cells [4-6]. Recent data suggests that E2-2 homodimers are critical for plasmacytoid dendritic cell development [7,8]. E2A also serves as an important dimerization partner for tissue specific HLH transcription factors outside of the lymphoid system. For example, E2A forms dimers with NeuroD and MyoD, key regulators of neuronal and skeletal muscle development, respectively [9-11]. Since E2A is the common factor for multiple lineage specific HLH transcription factor dimers, novel tools for targeting and manipulating E2A function can benefit a range of developmental biology research areas.

Here we have developed a new genetic model to examine E2A function. We have established an inducible E2A mouse model by inserting a tamoxifen-responsive region of the estrogen receptor (ER) ligand binding domain at the carboxyl end of *tcfe2a*, resulting in the production of E2AER fusion proteins. The use of tamoxifen inducible ER fusion proteins in mouse genetics has already been established as a valuable tool, especially with the vast use of the Cre recombinase-ER fusion protein for inducible gene knockout in mice [12-15]. In addition, ER fusion with a variety of transcription factors has also been successfully employed for analysis of gene expression. For example, MyoD-ER fusion proteins have been expressed by viral transduction in mouse embryonic fibroblasts for *in vitro *study of MyoD gene regulation [16] and in mouse fibroblasts for analysis of MyoD activation *in vivo *post transplantation of transduced cells into recipient animals [17]. However, we do not know how useful the ER system will be in live animals when targeting an endogenous locus. Our E2A^ER ^system now introduces an ER fusion with a bHLH transcription factor into the mouse genome for analysis of an endogenously expressed protein.

E2A^ER/+ ^and E2A^ER/ER ^mice, along with E2AER protein function, are analyzed here in the context of B cell development. A block in B cell development at the pre-proB cell stage, prior to B lineage commitment, has been characterized by previous E2A-deficient mouse models [18,19]. A rescue in B cell development from E2A^ER/ER ^pre-proB cells upon tamoxifen treatment would be a stringent test to verify inducible E2AER function.

In this study, we provide the initial characterization of the E2A^ER ^system. E2AER protein activity was rapidly induced upon tamoxifen treatment and reversibly regulated by tamoxifen withdrawal. Even though we show effective induction of E2AER DNA binding activity, tamoxifen treatment of E2A^ER/ER ^mice did not efficiently restore B cells *in vivo*. However, tamoxifen treatment was able to rescue and support early B cell development from E2A^ER/ER ^pre-proB cells in an *ex vivo *culture system. Use of E2A^ER ^*ex vivo *culture systems may therefore be beneficial for the study of gene regulation in B cells and other E2A-regulated cell lineages.

## Results

### Generation of the E2A^ER ^allele

**Figure 1:**
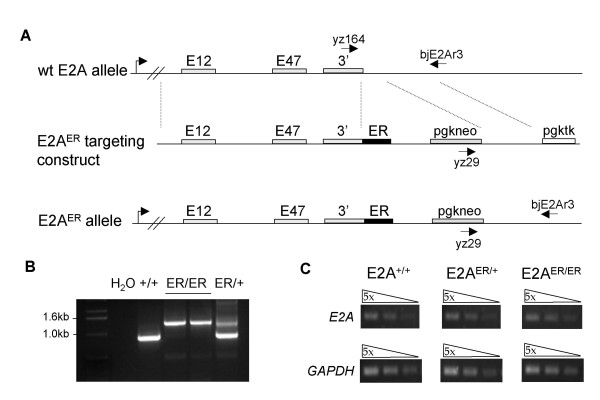
**Generation of the E2A^ER ^allele**. (A) Targeting strategy for the generation of E2A^ER/+ ^and E2A^ER/ER ^mice. The 3' region of the mouse *tcfe2a *gene was used for targeting. Gene direction, the E47, E12, and 3' exons (gray boxes), and inserted tamoxifen-responsive estrogen receptor ligand binding domain (ER, black box) are shown. Regions mediating homologous recombination are denoted by dotted lines. The selection markers pgkneo and pgktk are located as shown. (B) Genotyping PCR of E2A^+/+^, E2A^ER/+^, and E2A^ER/ER ^DNA using primers yz164, yz29, and bjE2Ar3 as shown in (A), that yield a 0.9 kb wild-type allele and 1.2 kb mutant allele. (C) RT-PCR of cDNA acquired from E2A^+/+^, E2A^ER/+^, and E2A^ER/ER ^thymus RNA. E2A and GAPDH (control) expression was detected by semi-quantitative PCR of 5-fold serial dilutions.

E2A^ER/+ ^and E2A^ER/ER ^mice were generated by using a knock-in strategy for tamoxifen-inducible E2A function. The tamoxifen-responsive ligand binding domain of the mouse ER [20] was inserted at the carboxyl end of *tcfe2a *to produce the E2A^ER ^allele (Figure 1A, B). With this targeting strategy, similar to that used for the E2A^GFP ^strain previously developed in our lab [21], both alternatively spliced products of the *tcfe2a *gene, E12 and E47, are translated as ER fusion proteins. Initial characterization of the E2A^ER ^allele indicated normal expression levels of E2A mRNA in the presence of the ER insertion (Figure 1C). Previous study of E2A knockout mice has demonstrated stunted growth and a high lethality rate of homozygous animals within the first few weeks after birth [18,19]. In contrast, the E2A^E47bm ^strain, expressing a dominant negative form of E47, was originally described as indistinguishable from wild-type litter mates in size and survival [22]. However, this work was analyzing mice on a mixed genetic background. Upon backcrossing to C57BL/6, the E2A^E47bm/E47bm ^mice became smaller in size and demonstrated the high lethality rate like that shown with the knockout animals (unpublished data). E2A^ER/ER ^animals also exhibit stunted growth and reduced survival (see Additional file 1). Fortunately, the lethality rate in our experience has been less severe in litters from the E2A^ER ^strain than that observed with our E2A knockout and dominant negative strains. However, we do not know if this slight increase in postnatal survival is due to the presence of the E2AER protein or because our E2A^ER ^strain is currently on a mixed background.

### The E2A^ER ^allele resembles an E2A-deficient allele in the absence of ligand

B cells develop from hematopoietic stem cells (HSC) in the bone marrow through a series of developmental stages [23]. The pre-proB cell stage is an intermediate stage as lymphoid progenitors develop into committed proB cells. Pre-proB cells can be characterized by the expression of B220 and CD43 and the absence of CD19 expression. As pre-proB cells transition to the proB cell stage, CD19 expression is induced and cells undergo commitment to the B cell lineage. E2A is critical for this transition, as demonstrated by the block in development at the pre-proB cell stage in E2A-deficient animals [18,19,24]. Analysis of E2A^GFP ^mice displays the up-regulation of E2A protein levels from the pre-proB to proB stage (see Additional file 2) [21]. This increase in E2A expression is likely critical for E2A's regulation of the B cell lineage gene expression profile given the importance of E2A gene dosage. For example, elimination of one copy of E2A has been shown to greatly reduce the number of proB cells [4,18].

**Figure 2:**
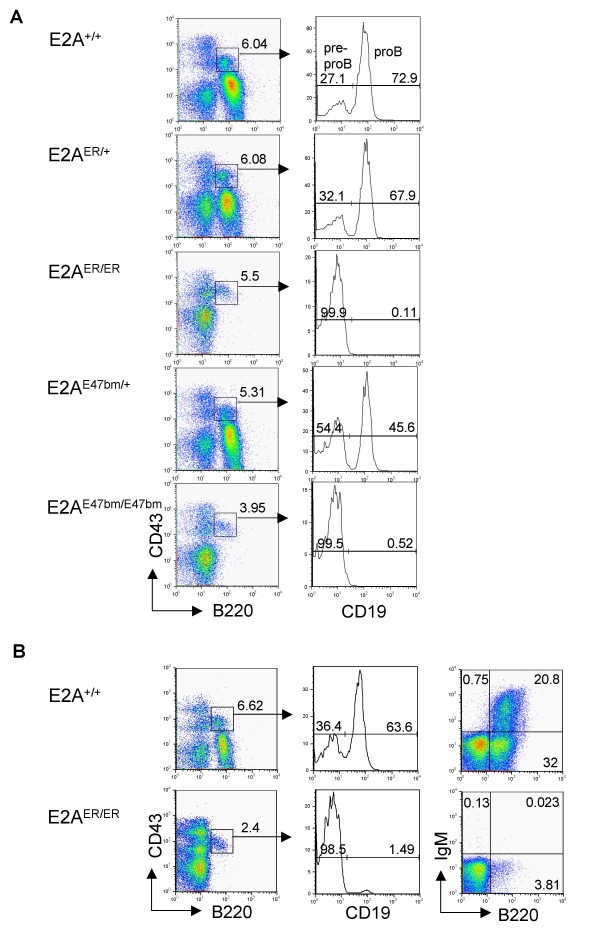
**B cell developmental block at the pre-proB cell stage in E2A^ER/ER ^mice**. (A) Representative staining of bone marrow cells from E2A^+/+^, E2A^ER/+^, E2A^ER/ER^, E2A^E47bm/+^, and E2A^E47bm/E47bm ^mice. Cells are pre-gated on 7AAD^- ^lymphocytes and relative percentages are given in each plot. Cells from the CD43^+^B220^+ ^gate are displayed in histograms analyzing CD19 expression. Pre-proB (CD19^-^) and proB (CD19^+^) cell percentages are shown. (B) Occasional CD19^+ ^B cell population detected in E2A^ER/ER ^mice. Staining of bone marrow from E2A^ER/ER ^and E2A^+/+ ^mice for B220, CD43, CD19, and IgM expression. All plots are pre-gated on 7AAD^- ^lymphocytes. Histograms are pre-gated on B220^+^CD43^+ ^cells as above and relative percentages are shown.

Investigation of B cell development in E2A^ER/ER ^mice revealed a block at the pre-proB cell stage, similar to that seen in the E2A-mutant E2A^E47bm/E47bm ^mice (Figure 2A) [22]. Occasionally we have observed a small population of CD19^+ ^B cells in the bone marrow of E2A^ER/ER ^mice, but these incidences of leaky B cell development did not appear to produce a significant population of mature, Ig expressing B cells (Figure 2B). Even though E2A has been suggested to influence proliferation in developing B cells [20,25-27], no significant difference in expansion at the pre-proB cell stage was observed by BrdU analysis of E2A^ER/ER ^and wild-type mice (see Additional file 3).

**Figure 3:**
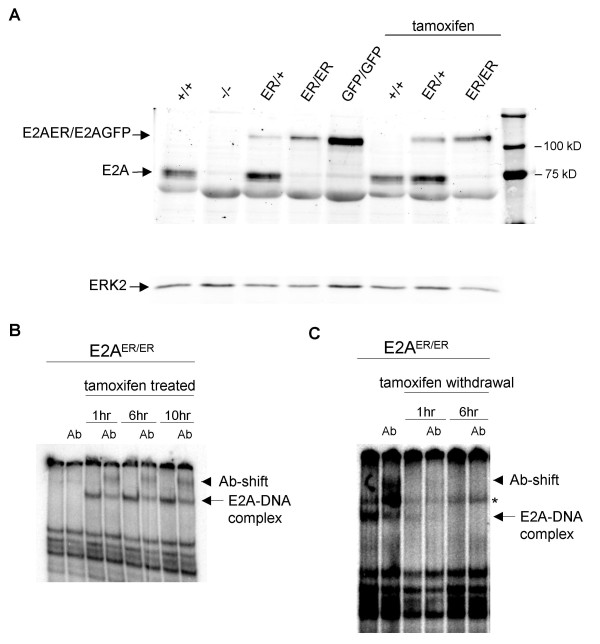
**Induction and reversible regulation of E2AER DNA binding activity**. (A) Detection of E2AER protein. Whole cell lysates were collected from E2A^+/+^, E2A^-/-^, E2A^ER/+^, E2A^ER/ER^, and E2A^GFP/GFP ^Abelson-transformed preB cell lines and analyzed by Western Blot for E2A protein expression using an anti-E2A antibody. E2A^+/+^, E2A^ER/+^, and E2A^ER/ER ^Abelson cells were also cultured with tamoxifen prior to analysis, as indicated. E2A^+/+^, E2A^GFP/GFP^, and E2A^-/- ^lines were used as positive and negative controls for E2A proteins. ERK2 was used as a loading control. (B and C) Analysis of E2AER DNA binding activity. E2A^ER/ER ^Abelson-transformed preB cells were cultured (B) without and with tamoxifen for 1, 6 and 10 hr and (C) with tamoxifen and upon tamoxifen withdrawal for 1 and 6 hr. Nuclear extracts were analyzed by gel shift for E2A DNA-binding using a μE5 probe. Anti-E2A antibody was used to demonstrate specificity (Ab). E2A-DNA complexes (arrow) and antibody-shifted complexes (arrow head) are indicated. *Non-specific band.

For analysis of E2AER protein expression and DNA binding activity, we chose to derive Abelson transformed preB cells from E2A^ER/+ ^and E2A^ER/ER ^bone marrow [28]. E2AER protein expression was verified by Western Blot analysis of whole cell lysates from E2A^ER/+ ^and E2A^ER/ER ^Abelson cells (Figure 3A). While E2AER protein levels appeared similar to wild-type levels when comparing E2A^+/+ ^and E2A^ER/ER ^cells, analysis of E2A^ER/+ ^cells suggested that E2AER protein expression may be lower than wild-type. Protein analysis of tamoxifen treated cells displayed similar results, indicating that the relative E2AER protein levels are not affected by the presence or absence of tamoxifen. Nuclear extracts from E2A^ER/ER ^Abelson cells were then used to conduct electrophoretic gel shift analysis of E2AER binding to an E2A binding sequence, μE5. In the absence of tamoxifen treatment, no DNA binding activity was observed from the E2AER protein (Figure 3B). Together, the block in B cell development and lack of E2AER DNA binding activity suggest that the E2A^ER ^mouse model functions as an E2A-deficient system in the absence of ligand.

### Rapid activation and reversible regulation of E2AER activity

Tamoxifen treatment of E2A^ER/ER ^Abelson cells resulted in rapid E2AER DNA binding activity within 1 hr of treatment (Figure 3B). The specificity of the protein binding to the μE5 probe was verified by using an anti-E2A antibody that effectively super-shifted the protein/DNA complex. The effect of tamoxifen withdrawal was then tested by washing tamoxifen-treated cells and growing them in the absence of tamoxifen for 1 and 6 hr time points. Loss of E2AER DNA binding activity was seen within 6 hrs of tamoxifen withdrawal (Figure 3C), indicating relatively fast reversibility of E2A function. These results demonstrate that the E2A^ER ^model can be used not only as an inducible model, but this system may also be valuable for providing a tightly regulated window of E2A activity.

### Induction of E2AER activity supports early B cell development *ex vivo*

**Figure 4:**
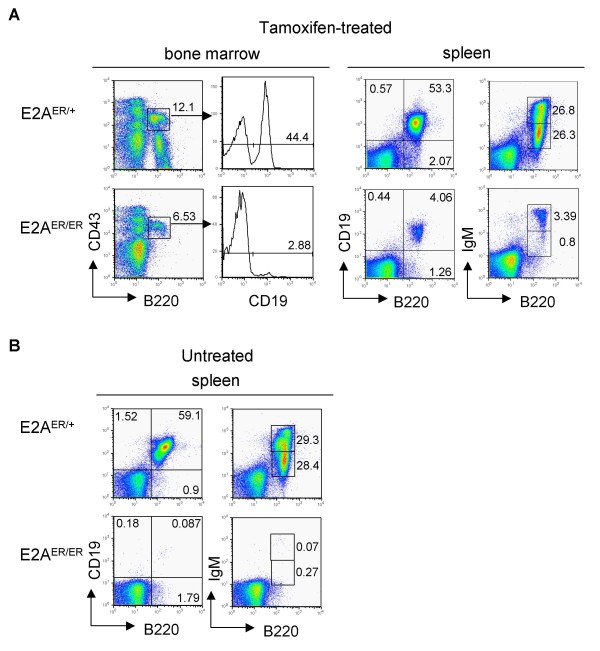
**Inefficient rescue of B cell development upon *in vivo *tamoxifen treatment of E2A^ER/ER ^mice**. (A) Two month old E2A^ER/+ ^and E2A^ER/ER ^control mice were treated with tamoxifen in their drinking water for 27 days. Indicated tissues were stained as shown. All plots are pre-gated on 7AAD^- ^lymphocytes. Histograms are pre-gated on B220^+^CD43^+ ^cells as shown. Relative percentages are displayed. (B) Representative spleen staining from age-matched non-treated E2A^ER/+ ^and E2A^ER/ER ^mice.

We first tested for a functional outcome of E2A induction by *in vivo *tamoxifen treatment of E2A^ER/ER ^mice followed by analysis of B cell populations in the bone marrow and spleen. All *in vivo *treatment efforts, including intraperitoneal injection and treatment in drinking water, unfortunately resulted in low efficiency rescue of B cell development. The emergence of B cells in tamoxifen-treated animals was rarely great enough to determine if the resulting B cells were generated in response to the tamoxifen treatment or were simply the incidence of leaky B cell development described above. The most significant recovery of B cells observed from *in vivo *treatment, which resulted from a 27 day tamoxifen treatment, was still considerably less than the B cell population in control mice (Figure 4A, B). However, the presence of Ig μ heavy chain (IgM) positive B cells in the spleen does suggest that a low level of tamoxifen-dependent B cell development occurred *in vivo*.

**Figure 5:**
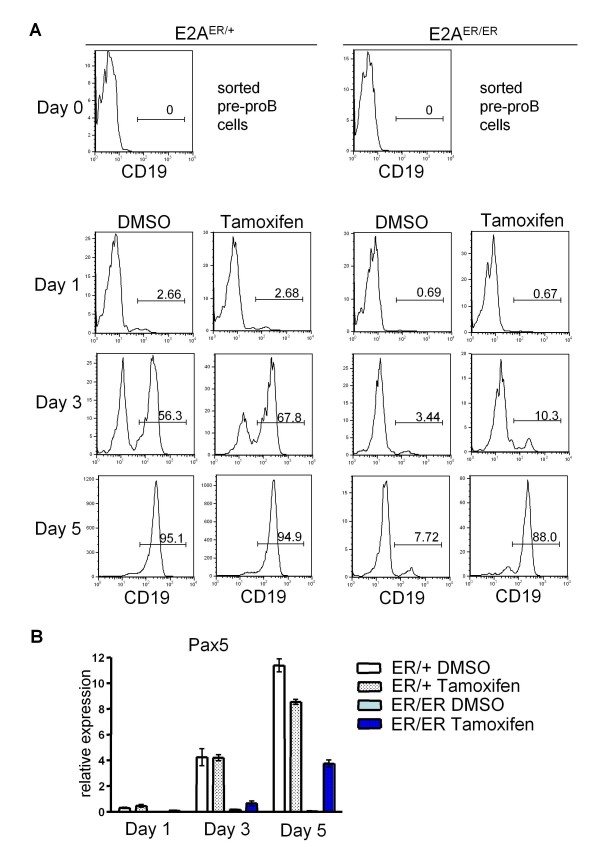
**Tamoxifen treatment restores B cell development from E2A^ER/ER ^pre-proB cells**. Sorted E2A^ER/+ ^and E2A^ER/ER ^pre-proB cells were plated in hormone-free media on S17 stromal cells on Day 0 in the presence of IL-7 with tamoxifen or DMSO (untreated control). (A) Cells are pre-gated on 7AAD^-^B220^+ ^lymphocytes. Percents of CD19^+ ^cells on Day 1, 3, and 5 are displayed. Data are representative of 4 independent experiments. (B) Expression of Pax5 was analyzed by quantitative RT-PCR from RNA collected from Day 1, 3, and 5 cultures shown in (A). Samples were normalized to the expression of GAPDH. Graphed results are means from triplicate runs (n = 3) with error bars representing standard error of the mean (SEM).

In contrast to *in vivo *tamoxifen treatment, treatment in an *ex vivo *B cell culture system effectively rescued the development of CD19^+ ^B cells. Sorted E2A^ER/ER ^pre-proB cells were cultured in hormone-free media on an S17 stromal layer in the presence of IL-7, with or without tamoxifen. Control DMSO treated E2A^ER/ER ^pre-proB cells failed to develop efficiently into CD19^+ ^B cells over the course of 5 days, whereas tamoxifen treated E2A^ER/ER ^pre-proB cells effectively gave rise to CD19^+ ^B cells (Figure 5A). Interestingly, the kinetics of B cell development from tamoxifen treated E2A^ER/ER ^pre-proB cells appeared delayed compared to that of control cells. In addition to using CD19 expression to validate the rescue of B cell development, we analyzed Pax5 expression throughout the 5 day culture. Pax5, initiated downstream of E2A expression, is a transcription factor critical for B cell lineage commitment [29]. Consistent with CD19 expression, induction of Pax5 expression was observed in tamoxifen treated E2A^ER/ER ^pre-proB cells, also appearing delayed compared to control cultures (Figure 5B).

**Figure 6:**
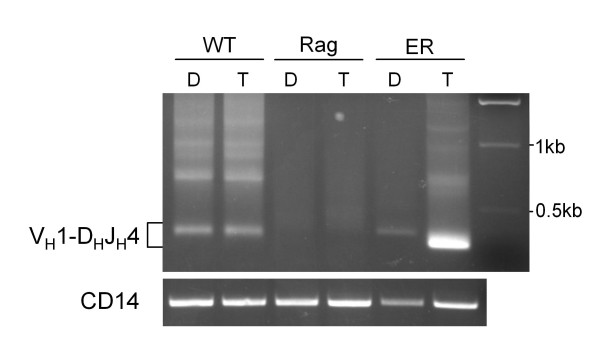
**IgH recombination detected upon culture of tamoxifen treated E2A^ER/ER ^pre-proB cells**. DNA was analyzed for IgH V to DJ rearrangements from Day 8 cultured wild-type (WT), Rag1^-/- ^(Rag) and E2A^ER/ER ^(ER) pre-proB cells using V_H_1 and J_H_4 primers. Cells were cultured in hormone-free media on S17 stromal cells with IL-7 and tamoxifen (T) or DMSO (D). V-DJ rearrangement products using V_H_1 family gene segments and J_H_4 are shown. WT and Rag were used as positive and negative controls, respectively. No products were detected when using dH_2_O as an additional negative control (data not shown). CD14 was used to demonstrate DNA loading. This result is representative of 4 independent nested PCR reactions.

Another key event during early B cell development, downstream of E2A function, is rearrangement of the Ig heavy chain locus (IgH) [2,18,19]. We next used this culture system to determine if induction of E2AER activity could also support IgH V to DJ recombination. To amplify V to DJ rearrangements, we chose primers recognizing members of the V_H_1 gene family [30], which represent a large percentage of the total IgH V genes, and primers recognizing the J_H_4 gene segment. Rearrangements were analyzed in Day 8 cultured samples because we did not observe consistent V to DJ PCR signals until this point in the time course analysis. Wild-type mice were used as positive controls for detecting recombination events, and mice deficient in the recombination activating gene RAG1, required for V(D)J recombination, were used as negative controls. Analysis of Day 8 cultures demonstrated a clear V to DJ rearrangement product from tamoxifen treated E2A^ER/ER ^cells (Figure 6). V to DJ rearrangement was further verified by sequencing analysis (see Additional file 4). The faint product detected from DMSO control treated E2A^ER/ER ^cells was accompanied by a small "leaky" CD19^+ ^population generated at this relatively late time point in the culture system (see Additional file 5). However, only one unique rearrangement product was identified out of all of the colonies sequenced for this sample. In addition to providing functional proof of induced E2A activity, the detection of IgH recombination, along with the induction of CD19 and Pax5 expression described above, suggests that restored E2A function can rescue B cell development from the pre-proB cell stage.

## Discussion

The E2A^ER ^system provides an improved method for analyzing E2A function. Previous analysis using over-expression of E2AER fusion proteins by retroviral transduction has already demonstrated the value of inducible E2A activity [20,31-34]. However, toxicity is often a problem with high levels of E2A in the cell [20,35], and viral transduction is not ideal for all cell types. In addition, the changes in gene expression detected upon over-expression of E2A may not always be representative of endogenous E2A function. Therefore, generation of the E2A^ER ^allele provides an attractive system for studying gene regulation and other E2A-regulated events in potentially any E2A-expressing cell type at a more physiological level.

Here we demonstrate the rapid induction of E2AER DNA binding activity upon tamoxifen treatment and its potential for reversible function upon tamoxifen withdrawal. The ability to tightly control E2A activity allows for kinetic analysis of downstream events. Analysis of B cell development in our *ex vivo *culture system demonstrated a rescue from the pre-proB cell stage, but suggested delayed kinetics from E2A^ER/ER ^pre-proB cells compared to B cell development from control pre-proB cells. This result suggests that even though E2A^ER/ER ^and wild-type pre-proB cells are phenotypically similar, E2A^ER/ER ^pre-proB cells may not have equivalent developmental potential or may require additional cellular changes prior to entering the B cell lineage. It is possible that E2A-regulated events normally occurring earlier in development, as suggested in hematopoietic stem cell (HSC), lymphoid-primed multipotent progenitor (LMPP), and common lymphoid progenitor (CLP) stages [27,36,37], were occurring upon restored E2A function in pre-proB cells and requiring these cells to "catch up" with their wild-type counterparts prior to progressing to the next stage. Alternatively, the developing tamoxifen-treated E2A^ER/ER ^B cells may not have expanded as rapidly as wild-type cells upon commitment to the B cell lineage. Our Western Blot analysis of E2A proteins in E2A^ER/+ ^Abelson cells (Figure 3A) suggested that the E2AER protein level may also have been contributing to the delayed kinetics. In this sample, the E2AER band appeared less intense than the wild-type E2A band. However, it is not possible to determine from this analysis if the lower intensity was a result of reduced protein levels or variation in antibody affinity for the E2AER versus wild-type E2A protein. In addition, there was not a striking difference between the E2AER and wild-type protein levels when comparing the E2A^+/+ ^and E2A^ER/ER ^samples. Further investigation of tamoxifen treated E2A^ER/ER ^pre-proB cells will be necessary to understand why the delayed detection of CD19^+ ^B cells was observed.

Our *ex vivo *culture data clearly indicated a rescue of early B cell development to the proB cell stage. However, we have so far been unable to determine if tamoxifen treatment can rescue E2A^ER/ER ^B cells through the subsequent preB cell stage, when E2A is known to be critical for Ig kappa light chain (Igk) recombination [38]. We were unable to test for induction of Igk recombination or surface IgM expression by using our *ex vivo *culture system because this system primarily supports development at a stage prior to these events (see Additional file 5 and data not shown). The presence of the small, but increased, population of E2A^ER/ER ^IgM^+ ^B cells in the *in vivo *treatment experiment (Figure 4A) is the only current evidence suggesting tamoxifen-induced E2AER activity may be able to rescue B cell development to maturity. Additional B cell culture systems would be necessary to determine if tamoxifen treatment can rescue later stages of E2A^ER/ER ^B cell development.

While *in vivo *tamoxifen treatment was not efficient for restoring B cell development, *in vivo *treatment may still be useful for analysis of E2A function in other cell lineages. We suggest two reasons for ineffective rescue of *in vivo *B cell development. First, progression through several stages of lymphocyte development is known to be dependent on proper E-protein dosage [4,6,18,26,39,40], and it is possible that the required E2A threshold is not maintained during our *in vivo *treatment for rescue of B cell development. For example, as mentioned above, we do not know if E2A^ER/ER ^cells can sufficiently progress through the preB cell stage of development. Second, E2A has been suggested to be important for B cell survival [41]. Therefore, it is conceivable that even if B cells are rescued by tamoxifen treatment, they may be lost if E2AER activity is not maintained throughout the treatment.

The E2A^ER ^system may instead be more valuable for *in vivo *study of T cell development, or other lineages expressing multiple E-protein family members. Since E-proteins demonstrate many redundant functions, T cell development is not completely blocked in E2A-deficient mice due to the presence of HEB [4,6,42,43]. Because T cells are fully developed in E2A-deficient mice, *in vivo *tamoxifen treatment of E2A^ER/ER ^mice may be more valuable for the study of E2A function during T cell development.

There are many additional applications for the E2A^ER ^system. In addition to advantages in speed, expression level, and reversibility, this inducible system may allow analysis of E2A function in specific cell stages that have been difficult to analyze in previous E2A-deficient models. Tamoxifen treatment of E2A^ER/ER ^cells may be valuable by allowing progression to developmental stages that are absent in E2A-deficient animals or affected due to the lack of E2A at earlier developmental stages. E2A^ER/ER ^mice also provide a useful tool for studying changes in E2A function with age or for analyzing E-protein function in combination with other genetic models. Given the rapid induction of E2AER DNA binding activity upon *in vitro *tamoxifen treatment, we believe that the use of E2A^ER ^*ex vivo *culture systems may be most valuable, especially for identifying new E2A targets and studying the kinetics of E2A gene regulation.

## Conclusion

We have established a new genetic model by generating the E2A^ER ^allele. This model allows for inducible function of E2A, a transcription factor displaying an extensive range of functions across multiple developmental programs. E2AER activity is rapidly induced at the protein level upon tamoxifen treatment and is reversible upon tamoxifen withdrawal. Tamoxifen treatment of E2A^ER/ER ^mice was, however, unable to efficiently restore wild-type levels of B cells. The functionality of E2AER was instead successfully verified by *ex vivo *tamoxifen treatment of E2A^ER/ER^ B cell progenitors. In this *ex vivo *culture system, induced E2AER protein function was able to rescue and support early B cell development. Thus, the E2A^ER ^model provides an attractive system to regulate and study E2A protein function, especially under *ex vivo *conditions where cells can gain sustained access to high levels of tamoxifen.

## Methods

### Mice

E2A^E47bm ^mice have been described previously [22]. Generation of the E2A^ER ^allele is described below. All research with mice was performed in accordance with relevant guidelines, and protocols were approved by the Duke University Animal Care and Use Committee.

### Generation of the E2AER allele

The gene targeting strategy used was a modification of the strategy for generation of the E2A^GFP ^strain [21]. The tamoxifen-responsive region of the mouse estrogen receptor ligand binding domain containing the G525R mutation [44] was PCR amplified from the MigR1-E47R vector [20] using the primers ERfpA: 5'-CGGATCCACGAAATGAAATGGGTGC-3' and ERrpA: 5'-CCGGCCGCTAGAATTCGATCGTGTTGGGGAAGCCCTC-3' to introduce a 5' BamHI site and 3' EcoRI and EagI sites for subsequent cloning steps. The ER fragment was inserted, replacing EGFP, at the BamHI position in frame with E2A. The targeting construct also contained a positive selection marker, PGKNeo cassette, and a negative selection marker, PGK driven thymidine kinase (TK) cassette. Mouse ES cells used were derived from a 129/sv strain obtained from Phillippe Soriano's lab in 1995 and then maintained in our own lab. E2A^ER/+ ^and E2A^ER/ER ^mice were maintained on a C57BL6 and 129/sv mixed background. Three primers were used for detection of wild-type and mutant alleles, yz164: 5'-AAGAACGAGGCCTTCCGTGTC-3', yz29: 5'-TCGCAGCGCATCGCCTTCTA-3', and bjE2Ar3: 5'-CAAGAGACTAGGATGCCACTG-3'.

### RT-PCR

RNA extraction, DNase I treatment and reverse transcription have been described previously [41]. Quantitative real-time PCR analysis for Pax5 expression was performed using a Roche LightCycler and Fast-Start DNA master SYBR green kit I (Roche) as per manufacturer's instructions. The following primers were used, E2A: f1 5'-CCAGTCTCAGAGAATGGCAC-3' and r1 5'-CCTTCGCTGTATGTCCGGCTAG-3'; Pax5 and GAPDH primers [41].

### Cell staining and flow-cytometry

For sorting, bone marrow was harvested and pooled from 2-3 mice per genotype. Cells positive for lineage markers Mac-1, Gr-1, Ter-119, and CD3 were depleted with Dynal Dynabeads (Invitrogen) according to manufacturer's instructions. Dead cells stained with 7-aminoactinomycin D (7AAD, Molecular Probes) were excluded. Pre-proB cells were further distinguished as B220^+^CD43^+^CD19^-^. FACS analysis was done with a FACSCalibur (BD Biosciences) or FACSVantage SE with DiVa option (BD Biosciences) and FlowJo software (Tree Star). FACSVantage SE with DiVa option was used for cell sorting.

### Tamoxifen preparation for *in vitro *culture treatment

Tamoxifen (Sigma) was prepared as a 1 mM stock (1000×) dissolved in cell culture grade dimethyl sulfoxide (DMSO) and stored at -20°C.

### Abelson transformed preB cells

The E2A^ER/ER ^Abelson preB cell line was derived by Abelson Murine Leukemia Virus transformation of bone marrow cells from an E2A^ER/ER^ mouse. Briefly, whole bone marrow was plated on an S17 stromal layer in the presence of 1 uM tamoxifen and 10 ng/mL IL-7 in 5% FBS RMPI media. This culture was performed prior to transduction to ensure cells were proliferating and were at the optimal target stage for Abelson transformation. Once an expanding B cell population was observed, cells were infected with Abelson virus in the presence of 4 ug/mL polybrene. Abelson transformed cells were then removed from the stromal layer, and tamoxifen and IL-7 were withdrawn. The established E2A^ER/ER ^Abelson preB cell line was maintained in 10% FBS RPMI media (also containing 100 units/ml penicillin, 100 ug/mL streptomycin and 55 uM 2-mercaptoethanol) prior to experimental analysis. Additional Abelson lines were established as described previously [32].

### Western Blot

Abelson preB cell lines were cultured without tamoxifen or with 1 uM tamoxifen for 24 hr prior to analysis. Cells were lysed in RIPA lysis buffer (1% Triton, 0.5% sodium deoxycholic acid, 0.1% SDS, 25 mM Tris-Cl pH 7.6, 150 mM NaCl, 5 mM EDTA) with protease inhibitors. Whole cell lysates were resolved by SDS-PAGE and blotted with anti-E2A (G127-32, BD Biosciences, 554077) and anti-ERK2 (C-14, Santa Cruz Biotechnology, sc-154) antibodies.

### Electrophoretic mobility shift assay

E2A^ER/ER ^Abelson preB cells were cultured with or without 1 uM tamoxifen as indicated. For withdrawal analysis, tamoxifen-treated cells were washed and re-plated in the absence of tamoxifen for the indicated times. Nuclear extracts were incubated with a ^32^P-labeled μE5 oligonucleotide probe, with or without Yae anti-E2A monoclonal antibody (Santa Cruz Biotechnology, sc-416), and resolved on a 5% polyacrylamide gel. Gels were dried and exposed to a phosphor screen for phosphorimager analysis (Amersham Biosciences). Oligos used for μE5 probe: 5'-TCGAAGAACACCTGCAGCAGCT-3' and 5'-TAGAGCTGCTGCAGGTGTTCTT-3'.

### *In vivo *tamoxifen treatment

Mice were treated with tamoxifen in the drinking water for 27 days. A 68 mg/mL tamoxifen in ethanol stock was used to bring the concentration in drinking water to approximately 26 ug/mL, resulting in 0.04% ethanol in water. A fresh bottle of tamoxifen water was given every 5 days.

### *Ex vivo *pre-proB culture system

Sorted pre-proB cells were plated on an S17 stromal layer in 24-well plates at approximately 1.5 × 10^4 ^cells per well and cultured with 5% FBS RPMI hormone-free media containing 10 ng/mL IL-7. Hormone free media consisted of phenol-red free RPMI 1640 supplemented with 5% charcoal/dextran treated FBS (Hyclone), 100 units/mL penicillin, 100 ug/mL streptomycin, and 55 uM 2-mercaptoethanol. Treated wells contained 1 uM tamoxifen and untreated controls were given DMSO alone (0.1%). Cells received fresh media, cytokine, and tamoxifen or DMSO every other day. Cells were harvested at time points indicated, and samples were split in half for FACS analysis and RNA or DNA extraction.

### IgH rearrangement analysis

Sorted pre-proB cells were *ex vivo *cultured as described above. DNA was extracted from Day 8 cultures and then analyzed for IgH V to DJ rearrangements using V_H_1 gene family and J_H_4 specific primers. A nested PCR strategy was used to amplify rearrangements involving V_H_1 family gene segments. The following primers were used for Round 1: V_H_1 ext 5'-AGRTYCAGCTGCARCAGTCT-3' [30] and J_H_4 YZB6 5'-TCCCTCAAATGAGCCTCCAAAGTCC-3' [45] and for Round 2: V_H_1 int 5'-GARGATRTCCTGYAAGGCTTC -3' [30] and J_H_4 YZB5 5'-CCTGAGGAGACGGTGACTGAGGTTCCTTG-3'[46]. CD14 primers were used to demonstrate the presence of DNA in all samples [41]. V_H_1-D_H_J_H_4 PCR products were cloned into a pCR4 TOPO vector (Invitrogen) and sequenced (Direct Sequencing from Colonies Service, Eton Bioscience, Inc). Rearrangement product sequences were analyzed by SoDA [47].

## Authors' contributions

MEJ participated in the design of the study, carried out the studies, and drafted the manuscript. MK participated in the design of the study and assisted with cell sorting. YZ conceived of the study and participated in its design. All authors read and approved the final manuscript.

## Supplementary Material

Additional file 1 **Characterization of E2A gene-targeted mice**. Phenotypes are descriptive of homozygous animals. Click here for file

Additional file 2 **Up-regulation of E2A protein levels during pre-proB to proB cell stage development**. (A) E2A^GFP ^allele. An E2A-GFP fusion protein is produced from this allele. Therefore, GFP expression can be used to monitor E2A protein levels. (B) E2A^GFP/GFP ^and E2A^+/+ ^control bone marrow was stained for B220, CD43, and CD19 surface expression. Cells are pre-gated on lymphocytes. GFP expression is shown for E2A^+/+ ^B220^+^CD43^+ ^(pre-proB + proB) control cells, and E2A^GFP/GFP ^pre-proB (B220^+^CD43^+^CD19^-^) and proB (B220^+^CD43^+^CD19^+^) compartments. Click here for file

Additional file 3 ***In vivo *BrdU labeling suggests similar expansion of E2A^ER/ER ^pre-proB cells compared to E2A^+/+ ^pre-proB cells**. Two mice from each genotype, E2A^ER/ER ^and E2A^+/+ ^control, were IP injected with 1 mg BrdU and analyzed 4 hrs post injection. Bone marrow was stained for B220, CD43, and CD19 surface expression, then processed to analyze BrdU labeling with a FITC BrdU Flow Kit as per manufacturer's instructions (BD Pharmingen). All plots are pre-gated on lymphocytes. Graphs display percent BrdU^+ ^cells within the pre-proB population (B220^+^CD43^+^CD19^-^) as labeled. An E2A^ER/ER ^mouse receiving no BrdU injection was used as a negative control (plot displays pre-proB cells). The proB population (B220^+^CD43^+^CD19^+^) from BrdU injected E2A^+/+ ^#2 is shown as a positive control for BrdU incorporation. Click here for file

Additional file 4 **Sequencing analysis of IgH V-DJ rearrangements**. PCR products from the IgH V to DJ rearrangement analysis in Figure 6 were sequenced for the following Day 8 cultured samples: E2A^+/+ ^(WT) tamoxifen treated, and E2A^ER/ER ^(ER) DMSO treated and tamoxifen treated. The total numbers of colonies sequenced per sample are shown. Sequences encoding V_H_1 to D_H_J_H_4 rearrangement products were determined. Unique rearrangement products were defined as products using a unique set of V, D, and J gene segments or containing a unique number of nucleotide additions/deletions. The average length of unique rearrangement products from the internal (Round 2) V_H_1 and J_H_4 primers is also shown for each sample analyzed. Click here for file

Additional file 5 **Phenotype of cultures utilized for IgH V-DJ rearrangement**. Staining of Day 8 cultures for CD19 and IgM expression. Cells are pre-gated on 7AAD^-^B220^+ ^lymphocytes. Relative percentages are displayed. Click here for file
