# Supplementary material for: A tamoxifen inducible knock-in allele for investigation of E2A function
Source: BMC Dev Biol. 2009 Oct 12;9:51. doi: 10.1186/1471-213X-9-51 (PMC2765948; doi:10.1186/1471-213X-9-51)
Supplement: Additional file 1 — Characterization of E2A gene-targeted mice. Phenotypes are descriptive of homozygous animals. [file 1471-213X-9-51-S1.PDF]

	$E2A^{+/+}$ wild type	$E2A^{gal}$ knockout null allele	$E2A^{E47bm}$ knockin basic region mutation	$E2A^{GFP}$ knockin C-term fusion	$E2A^{ER}$ knockin C-term fusion
**E2A activity**	normal	null	dominant negative	normal	tamoxifen inducible
**Growth**	normal	stunted	stunted	normal	stunted in most
**Viability**	normal	high rate lethality	high rate lethality	normal	moderate rate lethality
**B cell development**	normal	block at pre-proB to proB stage	block at pre-proB to proB stage	normal	block at pre-proB to proB stage

Additional file 1 – Characterization of E2A gene-targeted mice.
Phenotypes are descriptive of homozygous animals.
