# Supplementary material for: A tamoxifen inducible knock-in allele for investigation of E2A function
Source: BMC Dev Biol. 2009 Oct 12;9:51. doi: 10.1186/1471-213X-9-51 (PMC2765948; doi:10.1186/1471-213X-9-51)
Supplement: Additional file 2 — Up-regulation of E2A protein levels during pre-proB to proB cell stage development. (A) E2AGFP allele. An E2A-GFP fusion protein is produced from this allele. Therefore, GFP expression can be used to monitor E2A protein levels. (B) E2AGFP/GFP and E2A+/+ control bone marrow was stained for B220, CD43, and CD19 surface expression. Cells are pre-gated on lymphocytes. GFP expression is shown for E2A+/+ B220+CD43+ (pre-proB + proB) control cells, and E2AGFP/GFP pre-proB (B220+CD43+CD19-) and proB (B220+CD43+CD19+) compartments. [file 1471-213X-9-51-S2.PDF]

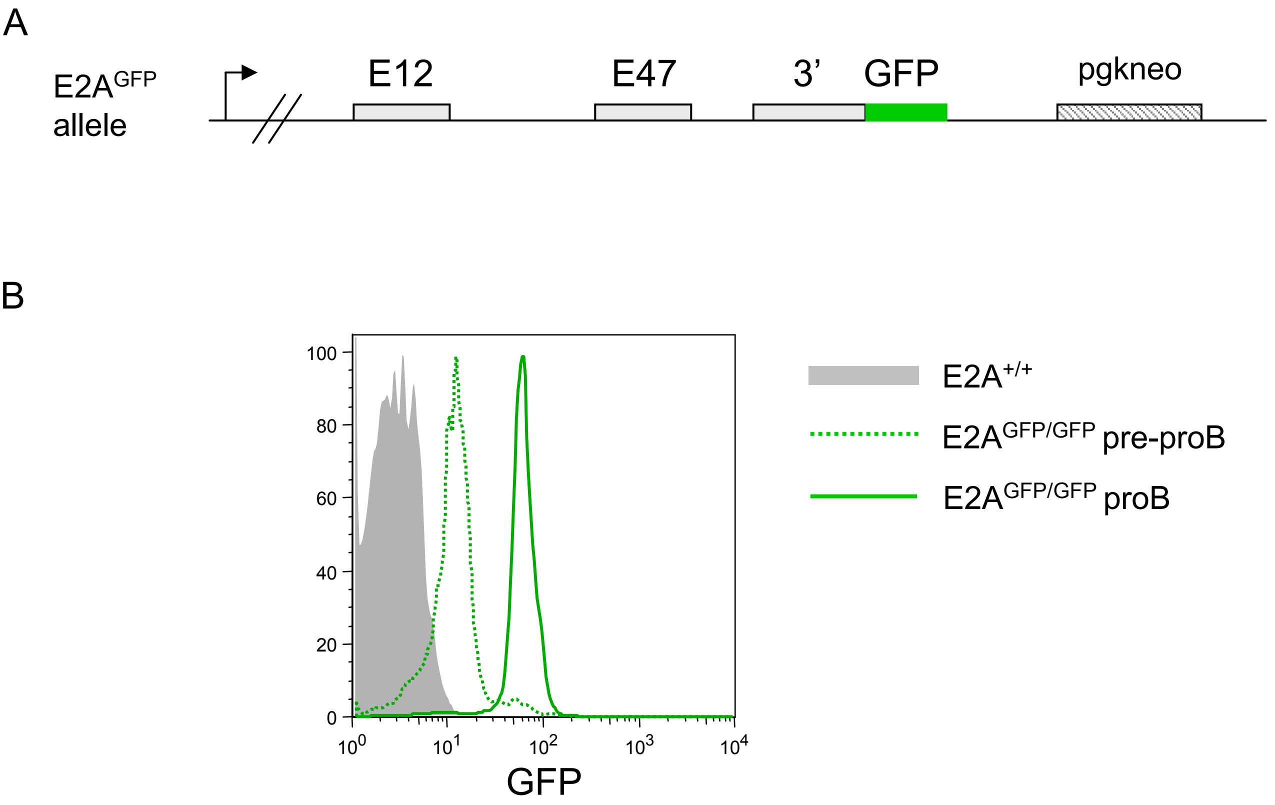

Additional file 2 – Up-regulation of E2A protein levels during pre-proB to proB cell stage development. (A) $E2A^{GFP}$ allele*. An E2A-GFP fusion protein is produced from this allele. Therefore, GFP expression can be used to monitor E2A protein levels. (B) $E2A^{GFP/GFP}$ and $E2A^{+/+}$ control bone marrow was stained for B220, CD43, and CD19 surface expression. Cells are pre-gated on lymphocytes. GFP expression is shown for $E2A^{+/+}$ $B220^{+}CD43^{+}$ (pre-proB + proB) control cells, and $E2A^{GFP/GFP}$ pre-proB ($B220^{+}CD43^{+}CD19^{-}$) and proB ($B220^{+}CD43^{+}CD19^{+}$) compartments.

*Zhuang Y, Jackson A, Pan L, Shen K, Dai M: **Regulation of E2A gene expression in B-lymphocyte development.** *Mol Immunol* 2004, **40**:1165-1177.
