# Supplementary material for: A tamoxifen inducible knock-in allele for investigation of E2A function
Source: BMC Dev Biol. 2009 Oct 12;9:51. doi: 10.1186/1471-213X-9-51 (PMC2765948; doi:10.1186/1471-213X-9-51)
Supplement: Additional file 3 — In vivo BrdU labeling suggests similar expansion of E2AER/ER pre-proB cells compared to E2A+/+ pre-proB cells. Two mice from each genotype, E2AER/ER and E2A+/+ control, were IP injected with 1 mg BrdU and analyzed 4 hrs post injection. Bone marrow was stained for B220, CD43, and CD19 surface expression, then processed to analyze BrdU labeling with a FITC BrdU Flow Kit as per manufacturer's instructions (BD Pharmingen). All plots are pre-gated on lymphocytes. Graphs display percent BrdU+ cells within the pre-proB population (B220+CD43+CD19-) as labeled. An E2AER/ER mouse receiving no BrdU injection was used as a negative control (plot displays pre-proB cells). The proB population (B220+CD43+CD19+) from BrdU injected E2A+/+ #2 is shown as a positive control for BrdU incorporation. [file 1471-213X-9-51-S3.PDF]

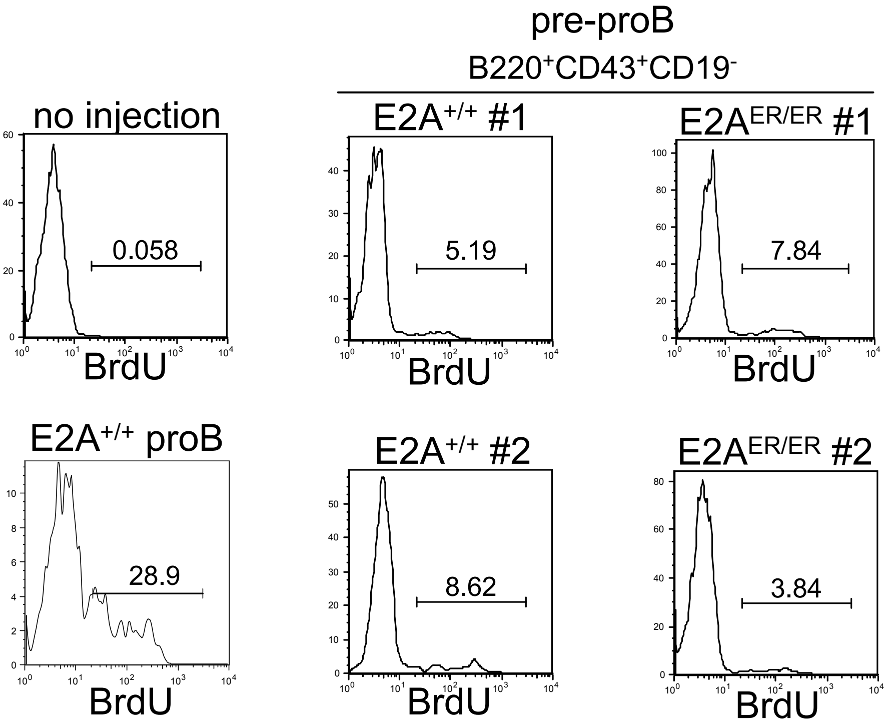

Additional file 3 – *In vivo* BrdU labeling suggests similar expansion of E2A$^{ER/ER}$ pre-proB cells compared to E2A$^{+/+}$ pre-proB cells. Two mice from each genotype, E2A$^{ER/ER}$ and E2A$^{+/+}$ control, were IP injected with 1mg BrdU and analyzed 4hrs post injection. Bone marrow was stained for B220, CD43, and CD19 surface expression, then processed to analyze BrdU labeling with a FITC BrdU Flow Kit as per manufacturer's instructions (BD Pharmingen). All plots are pre-gated on lymphocytes. Graphs display percent BrdU^{+} cells within the pre-proB population (B220^{+}CD43^{+}CD19^{-}) as labeled. An E2A$^{ER/ER}$ mouse receiving no BrdU injection was used as a negative control (plot displays pre-proB cells). The proB population (B220^{+}CD43^{+}CD19^{+}) from BrdU injected E2A$^{+/+}$ #2 is shown as a positive control for BrdU incorporation.
