# Supplementary material for: A tamoxifen inducible knock-in allele for investigation of E2A function
Source: BMC Dev Biol. 2009 Oct 12;9:51. doi: 10.1186/1471-213X-9-51 (PMC2765948; doi:10.1186/1471-213X-9-51)
Supplement: Additional file 4 — Sequencing analysis of IgH V-DJ rearrangements. PCR products from the IgH V to DJ rearrangement analysis in Figure 6 were sequenced for the following Day 8 cultured samples: E2A+/+ (WT) tamoxifen treated, and E2AER/ER (ER) DMSO treated and tamoxifen treated. The total numbers of colonies sequenced per sample are shown. Sequences encoding VH1 to DHJH4 rearrangement products were determined. Unique rearrangement products were defined as products using a unique set of V, D, and J gene segments or containing a unique number of nucleotide additions/deletions. The average length of unique rearrangement products from the internal (Round 2) VH1 and JH4 primers is also shown for each sample analyzed. [file 1471-213X-9-51-S4.PDF]

Day 8 Culture Sample	total # colonies sequenced	# sequences containing a V-D-J product	# unique V-D-J products	average nucleotide length of unique V-D-J products
WT (Tamoxifen)	7	7	7	297
ER (DMSO)	9	1	1	366
ER (Tamoxifen)	15	15	7	301

Additional file 4 – Sequencing analysis of IgH V-DJ rearrangements.
PCR products from the IgH V to DJ rearrangement analysis in Figure 6 were sequenced for the following Day 8 cultured samples: $E2A^{+/+}$ (WT) tamoxifen treated, and $E2A^{ER/ER}$ (ER) DMSO treated and tamoxifen treated. The total numbers of colonies sequenced per sample are shown. Sequences encoding V_H1 to D_HJ_H4 rearrangement products were determined. Unique rearrangement products were defined as products using a unique set of V, D, and J gene segments or containing a unique number of nucleotide additions/deletions. The average length of unique rearrangement products from the internal (Round 2) V_H1 and J_H4 primers is also shown for each sample analyzed.
