# Supplementary material for: A tamoxifen inducible knock-in allele for investigation of E2A function
Source: BMC Dev Biol. 2009 Oct 12;9:51. doi: 10.1186/1471-213X-9-51 (PMC2765948; doi:10.1186/1471-213X-9-51)
Supplement: Additional file 5 — Phenotype of cultures utilized for IgH V-DJ rearrangement. Staining of Day 8 cultures for CD19 and IgM expression. Cells are pre-gated on 7AAD-B220+ lymphocytes. Relative percentages are displayed. [file 1471-213X-9-51-S5.PDF]

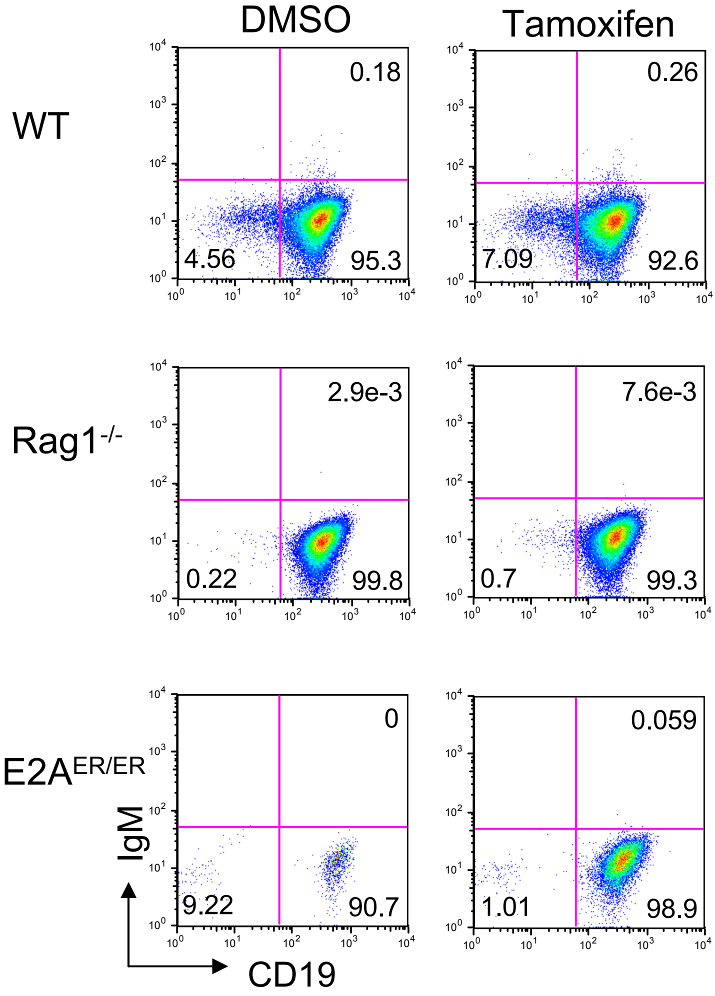

Additional file 5 – Phenotype of cultures utilized for IgH V-DJ rearrangement analysis. Staining of Day 8 cultures for CD19 and IgM expression. Cells are pre-gated on $7AAD^{-}B220^{+}$ lymphocytes. Relative percentages are displayed.
